# Computational Model for Predicting the Relationship Between Micro-RNAs and Their Target Messenger RNAs in Breast and Colon Cancers

**DOI:** 10.1177/1176935118785145

**Published:** 2018-07-02

**Authors:** Shinuk Kim

**Affiliations:** Department of Civil Engineering, Sangmyung University, Cheonan, Republic of Korea

**Keywords:** miRNA- mRNA relationship, computational method, cancer data

## Abstract

**Motivation:**

Uncovering the relationship between micro-RNAs (miRNAs) and their target messenger RNAs (mRNAs) can provide critical information regarding the mechanisms underlying certain types of cancers. In this context, we have proposed a computational method, referred to as prediction analysis by optimization method (PAOM), to predict miRNA-mRNA relations using data from normal and cancer tissues, and then applying the relevant algorithms to colon and breast cancers. Specifically, we used 26 miRNAs and 26 mRNAs with 676 (= 26 × 26) relationships to be recovered as unknown parameters.

**Results:**

Optimization methods were used to detect 61 relationships in breast cancer and 32 relationships in colon cancer. Using sequence filtering, we detected 18 relationships in breast cancer and 15 relationships in colon cancer. Among the 18 relationships, *CD24* is the target gene of let-7a and miR-98, and *E2F1* is the target gene of miR-20. In addition, the frequencies of the target genes of miR-223, miR-23a, and miR-20 were significant in breast cancer, and the frequencies of the target genes of miR-17, miR-124, and miR-30a were found to be significant in colon cancer.

**Availability:**

The numerical code is available from the authors on request.

## Introduction

Micro-RNAs (miRNAs) are a large class of small, non-coding RNAs consisting of 22 nucleotides that are expressed from longer and endogenous hairpin-shaped transcripts generally referred to as pre-miRNAs.^1^ The miRNAs regulate protein-coding gene expression post-transcriptionally via the translational repression or transcript degradation of their target messenger RNAs (mRNAs),^1,2^ thereby indicating that miRNAs perform crucial roles in a variety of biological functions. The results of recent studies have demonstrated that miRNAs are deregulated in cancers.^2,3^ Several computational methods have been proposed to determine how miRNAs pair with their target mRNAs,^3,4^ in an effort to unravel the roles of miRNAs in the deregulated expression of their target mRNAs during cancer development^5,6^ and some relationships were identified between miRNAs and their target mRNAs.^5,7–11^ However, the development of a computational method for the identification of such relationships in cancer remains a difficult issue. Thus far, 2 computational methods have been developed: either the identification of miRNAs that are conserved in different species or stem loop prosecutors^11^ or the identification of the relationship between miRNAs and their target mRNAs via the use of sequence homologues. Previously, we proposed a numerical optimization method for multi (miRNAs) -to -multi (mRNAs), which was used for the identification of 16 miRNAs-mRNA relations in colon cancer microarray profiles.^7^ The proposed method was used to identify 207 relationships successfully, out of 484. Some relationships detected in that study were verified through previous experimental evidences. For example, the relationship between miR-17 and its target E2F1 was identified in a previous colon cancer study.^12^ Like previous studies, however, the difference between the expression profiles of normal and cancer tissues, which we believe to be a critical factor in determining the relationship between miRNAs and their target mRNAs, was ignored.

Here, we propose a novel method, referred to as prediction analysis by optimization method (PAOM), which is composed of a mathematical model and computational method designed to predict miRNA-mRNA relations in the context of cancer development. In this model, the predicted relations are filtered using sequence analysis resources. For mathematical modeling, we employed linear system equations to obtain the inhibiting parameters. The role of the computational method is to optimize the relations and to allow for comparisons of the differences in the parameters between normal and cancer genes. We employed 2 optimization methods—the Broyden-Fletcher-Goldfarb-Shannon (BFGS) and the Powell method,^13^ both of which are well known for the optimization of a multi-dimensional matrix problem. For filtering sequence analysis, we used PicTar, based on the scanning multiple alignment of 3′ UTR (untranslated region) sequences and a search set of miRNA sequences, and scored the overlapping position.^11^ MiRanda is a miRNA target prediction algorithm that optimizes sequence complementarity using position-specific rules and relies on strict interspecies conservation requirements.^14^ In this study, we considered breast and colon cancer data with 676 relationships between 26 miRNAs and 26 mRNAs.

## Methods

The mathematical formulation and computational method of the PAOM are described in this section.

### Mathematical formulation

It is generally accepted that miRNAs regulate gene expression via either the transcript cleavage or translational repression of their specific target mRNAs, whereas 1 mRNA expression is regulated by multiple miRNAs. For this mechanism, we have a linear equation model, in which 1 mRNA is affected by several miRNAs $\left(x_{1},x_{2},\ldots ,x_{m}\right)$ as follows:

(1) $$y_{i}=a_{i1}x_{1}+a_{i2}x_{2}+\cdots +a_{im}x_{m},\;i=1,\ldots ,n$$

where $a_{ij}$ represents the influence of the *j*th miRNA on the *i*th mRNA, $x_{j}$ represents the expression level of the *j*th miRNA, and $y_{i}$ represents the expression level of the *i*th mRNA. To observe the relation between *m* miRNAs and *n* mRNAs simultaneously, we rewrite the system of linear equations in matrix form.

(2) $$\left[\begin{array}{c} y_{1}\\ y_{2}\\ \vdots \\ y_{n} \end{array}\right]=\left[\begin{array}{cccc} a_{11} & a_{12} & \cdots & a_{1m}\\ a_{21} & a_{22} & \cdots & a_{2m}\\ \vdots & \vdots & \ddots & \vdots \\ a_{n1} & a_{n2} & \cdots & a_{nm} \end{array}\right]\left[\begin{array}{c} x_{1}\\ x_{2}\\ \vdots \\ x_{m} \end{array}\right],$$

in which the measurements of the expression of *m* different miRNAs are denoted by $\left(x_{1},\ldots ,x_{m}\right)$ and of the expression of *n* different mRNAs are denoted by $\left(y_{1},\ldots ,y_{n}\right)$. From *K* times experiments,

(3) $$Y_{n\times K}=A_{n\times M}X_{m\times K}$$

We solve the equation as an inverse problem, and then obtain $a_{ij}$ as an unknown parameter, where $a_{ij}$ mainly represents the effect of $x_{i}$ and $y_{j}$, even if $a_{ij}$ is affected by the levels of other $x_{i}^{’}s(i’\neq i)$.

### Computational method

**Figure 1.**
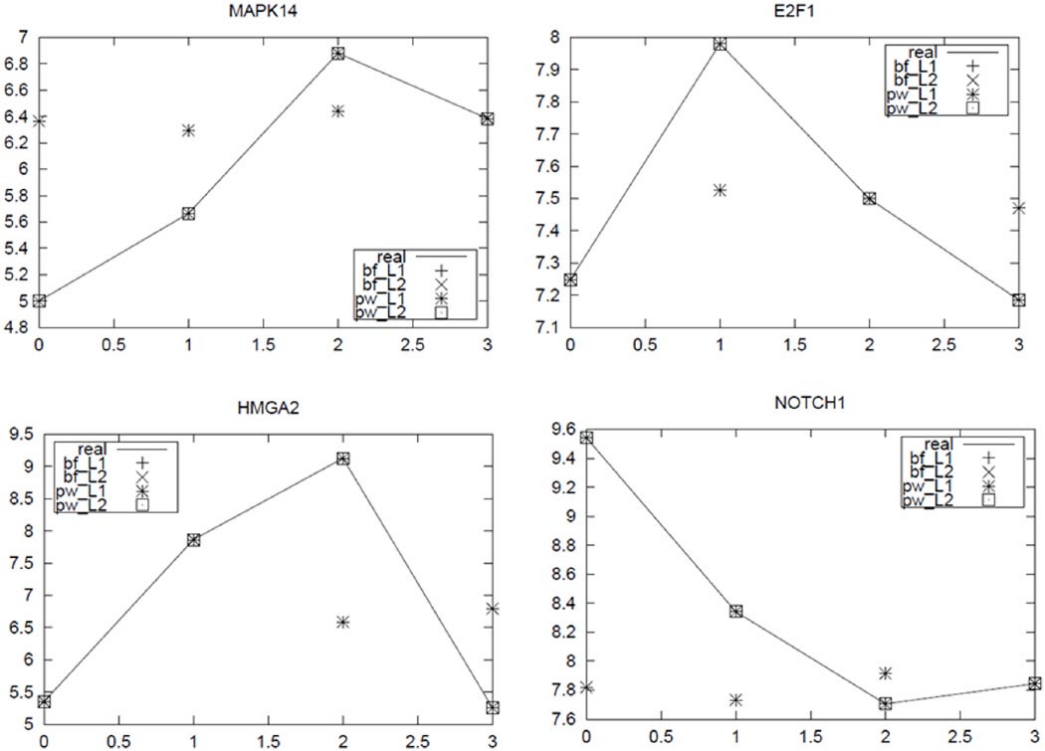
Comparison of real data set with reconstructed data set using obtained parameters from Powell and Broyden-Fletcher-Goldfarb-Shannon methods with $L_{1}$, and $L_{2}$ norms.

In this section, we present a computational scheme for the identification of the relationships between miRNAs and their target mRNAs. The computational method is composed of 3 components—the optimization routine, the objective function, and the direct code. Among them, the optimization routine is the principal component of the computational method, the role of which is to obtain a new set of parameter estimations by solving the inverse problem shown in Equation 3. In particular, we employ the BFGS method for optimization, the so-called quasi-Newton method, which requires second derivatives of the objective function and thereby makes a quadratic convergent to the minimum of error norm, coupled with a drastic reduction of the computational burden. The second method is the Powell method known as direction set method, in which no such second derivatives of the objective function are required. Both methods are useful for multi-dimensional optimization, but they do not work successfully in all cases. After testing both methods with small nodes, we selected an appropriate one for the current cases. Next, the objective function is to provide the criterion for further processing to the next iteration on the basis of iterative error norms. Regarding the error norm, we employed the $L_{1}$ and $L_{2}$ norms, which are as follows:

$$\begin{array}{l} L_{1}=\left|y\left(x,a_{ij}\right)-y_{real}\right|\\ L_{2}={\left(y\left(x,a_{ij}\right)-y_{real}\right)}^{2} \end{array}$$

where $y(x,a_{ij})$ is numerically computed data and $y_{real}$ is the experimentally obtained expression data. The $L_{1}$ norm worked successfully for the BFGS method and the $L_{2}$ norm worked better for Powell method. Finally, the direct solver is used to generate the computational data by solving the proposed mathematical model. Those computational data are then compared with the experimental data in an objective function. As both cancer and normal data were employed in this study, a note was that the predicted relations using cancer data are called $P_{cancer}$, and the predicted relations using normal data are called $P_{normal}$. Then, the relationships between the cancer and normal data sets were computed and compared with each other. Therefore, the numerical scheme is composed of 3 individual algorithms, such as 2 subroutines and 1 main routine, as shown in Figure 1. In the main routines, we compared the inhibitory relations of the normal data with the inhibitory relations of the cancer data, and then calculated the comparison values (CVs) using relative error as follows:

$$CV=\frac{\left(P_{normal}-P_{cancer}\right)}{\left|P_{normal}\right|}$$

As CV becomes bigger, the relation is proportional to the strength of the relation between mRNA and miRNA. With the normal and cancer expression data sets, we calculated the unknown parameters as inhibitory relations in the subroutine. The overall numerical scheme of the proposed algorithm follows.

### Data setting

We extracted experimentally known 26 miRNAs and 26 mRNAs from the RNA expression profiles of human cancers reported by Lu et al.^15^ In colon cancers, each gene consists of 4 normal and 7 cancer data points. In breast cancer, each gene consists of 3 normal and 6 cancer data points. As the data sets are much smaller than unknown parameters, malpositioning problem frequently occurs in the algorithm. For filtering analysis, we used PicTar (https://pictar.mdc-berlin.de/) and miRanda (http://www.microrna.org). PicTar is a computational method used to identify common targets of miRNAs, based on scanning multiple alignments of 3′ UTR sequences and a search set of miRNA sequences, followed by the scoring of the overlapping positions combining the PicTar scores of orthologous transcripts.^11^ miRanda is a miRNA target prediction algorithm that optimizes sequence complementarity using position-specific rules and relies on strict interspecies conservation requirements.^14^ Neither sequence filtering has any relation with cancers.

Main routine

Main input: data sets

1. Call subroutine

Input : miRNA cancer, miRNA normal mRNA cancer, and mRNA normal data sets.

Output: $P_{normal}\cdot P_{cancer}$

2. Compare the values of parameters

$\frac{\left(P_{normal}-P_{cancer}\right)}{\left|P_{normal}\right|}>0.5$

Output represents the relationship between miRNAs and their target mRNAs

Subroutine

Set $gtol=10^{-14}$

Read experimental data of the expression profiles of mRNAs and miRNAs;

1. Set initial guesses to zero
2. Construct linear model of Equation 2, and generate numerical data using miRNAs:D
3. Run Optimization method (BFGS)
4. Read expression microarray data profiles (normal:N or cancer:C)
5. Implement Objective function: f(err)=|D−N(orC)|

Iff(err)>gtol then go to 2

Iff(err)<gtol then

Output

$P_{normal}$: Parameters from normal data

$P_{cancer}$: Parameters from cancer data

## Results

**Figure 2.**
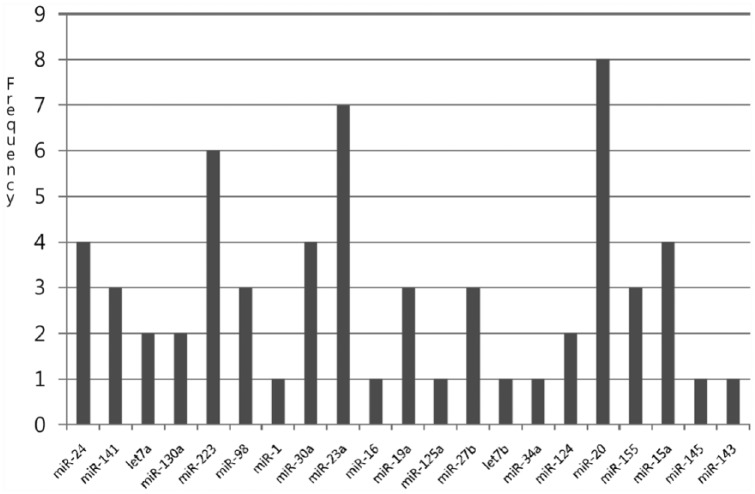
Distribution of the number of mRNAs of each miRNA with 5 PAOM in breast cancer. The most significant genes are *miR-23a, miR-223*, and *miR-20*. mRNA indicates messenger RNA; miRNA, micro-RNA; PAOM, prediction analysis by optimization method.

Figure 1 represents the comparisons of the real and reconstructed data based on the parameters derived from the Powell and BFGS methods. We tested 2 optimization methods: Powell and BFGS, with 4 nodes of mRNAs (MAPK14, E2F1, HMGA2, and NOTCH1) and 4 nodes of miRNAs (miR-34a, miR-17a, miR-24, and miR-30) from data points of the normal colon tissues. We also assessed each method with the *L*_1_ and *L*_2_ objective functions. The BFGS method with the $L_{1}$ and $L_{2}$ norms successfully reconstructed the real data points. The Powell method worked well with the $L_{2}$ norm for the reconstruction but not with $L_{1}$, which suggests that more numerical studies will be necessary to evaluate the performance of Powell method. When the BFGS method with the $L_{1}$ and $L_{2}$ norms was used for large nodes in the normal breast data, an error term of 0.011 was obtained with $L_{2}$ norm, thereby indicating that its accuracy was quite poor as compared with that of 5.97E-7 with the $L_{1}$ norm. In this study, therefore, we employed the BFGS method with $L_{1}$. Figure 2 shows the frequency of negative relationships with each of the presented miRNAs in breast cancer by cutting the PAOM score 5.

**Figure 3.**
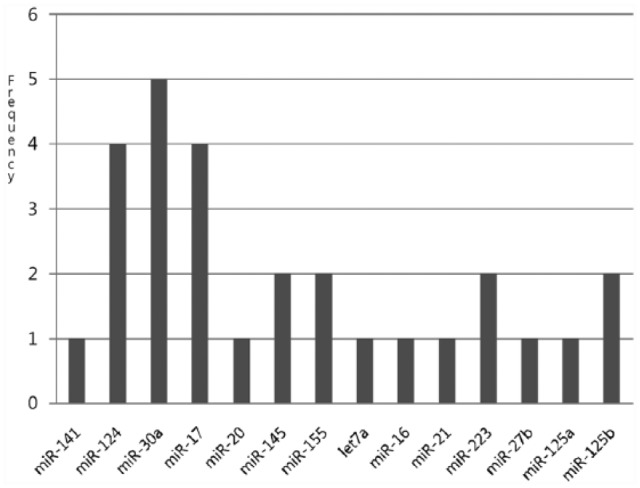
Distribution of the number of mRNAs of each miRNA with 5.0 PAOM in colon cancer. *miR-124, miR-30a*, and *miR-17* are the most significant. mRNA indicates messenger RNA; miRNA, micro-RNA; PAOM, prediction analysis by optimization method.

The frequency of miR-20, miR-23a, and miR-223 are relatively high, which suggests that those miRNAs do exert some effect on breast cancer.^16,17^ Zhang et al^6^ presented miR-20 with the copy number lost in breast cancer. Figure 3 represents the frequency of negative relation with each of the colon cancer miRNAs by cutting PAOM score 5. The frequencies of miR-17, miR-30a, and miR-124 were highest, which implies that those miRNAs exert an effect on colon cancer. Recently, Monzo et al^12^ observed that miR-17 was detected in human colon cancer development, and Silber et al^18^ observed upregulated miR-124 in colon (HCT-116) cancer cell lines. Tables 1 and 2 show the miRNAs-mRNAs relationships using the proposed method with filtering sequence analysis.

**Table 1.** The relation of miRNAs and target mRNAs with sequence analysis of breast cancer, experimental analysis, and optimization analysis.

mRNA	miRNA	miRandaa	PicTar^a^	PAOM^b^
MAPK14	miR-124	O	o	5.5
CLOCK	miR-141	O		7.9
NF2	miR-141	O		7.6
	miR-23a	O		5.0
	miR-27a	O		6.0
	miR-15	O	o	56.4
NFIA	miR-155	O		41.5
HMGA2	miR-20	O	o	10.5
THBS1	miR-19a	O		25.5
CXCL12	miR-141		o	8.3
E2F1	miR-20	O	o	57.3
NOTCH1	miR-155	O		8.5
SERP1	miR-223	O		5.5
CD24	let-7a	O		48.2
	miR-98a	O		12.0
POLR2	miR-223	O		6.1
	miR-98a	O		24.0
	miR-16	O		5.1

Abbreviations: mRNA, messenger RNA; miRNA, micro-RNA; PAOM, prediction analysis by optimization method.

a Predicted targets based on sequence analysis.

b Predicted targets based on the proposed method.

**Table 2.** The relation of miRNAs and target mRNAs with sequence analysis, experimental analysis, and the proposed optimization analysis based on colon cancer.

mRNA	miRNA	miRanda^a^	PicTar^a^	PAOM^b^
MAKP14	miR-141	O		8.9
	miR-124	O	o	6.6
CLOCK	miR-30a	O	o	15.2
	miR-17	O	o	10.0
	miR-20	O	o	5.0
HMGA2	miR-30a	O	o	5.0
THBS1	miR-155	O		7.5
CXCL12	miR-124	O		6.1
HRAS	let-7a	O		8.7
SIP1	miR-21	O		7.5
NOTCH1	miR-155	O		5.1
MTPN	miR-223	O	o	5.3
E2F3	miR-124	O		11.1
SERP1	miR-223	O		12.5
DVL2	miR-125a	O		20.1

Abbreviations: mRNA, messenger RNA; miRNA, micro-RNA; PAOM, prediction analysis by optimization method.

a Predicted targets based on sequence analysis.

b Predicted targets based on the proposed methods.

### Prediction of breast cancer

Table 1 shows the predicted relationships between miRNAs and mRNAs with sequence analysis in breast cancer. Based on our proposed method, we acquired 61 strong candidates out of a total of 676 miRNA-mRNA relations. With the integration of filtering sequence analysis, we predicted 18 miRNA-mRNA relationships in the breast cancer cells. Filtering analysis include sequence conservation-based miRanda and PicTar applications.

Those filtering resources are not generally associated with breast cancer. However, the current methods predicted some relationships using normal breast and cancer breast expression data. The results of our proposed method indicate that let-7a, miR-223, miR-98a, and miR-34a downregulate CD24 expression in cases of breast cancer. With sequence analysis, we suggest that let-7a (PAOM score 48.2) and miR-98a (PAOM score 12.0) are predicted to be strongly associated with CD24 in breast cancer. Those relationships were experimentally verified.^19,20^ Kaipparettu et al^19^ found that CD24 expression was downregulated by estrogen in breast cancer stem cells. Verghese et al^20^ found that let-7 family was downregulated significantly in breast tumor-initiating cells (BT-1Cs) that were enriched with CD24. Dai et al^21^ showed that *NF2* was a tumor suppressor gene in human breast cancer. The findings of the current study reveal that miR-24, miR-141, miR-23a, miR-19a, miR-27a, and miR-15a are involved in the downregulation of NF2 expression in human breast cancer cells. With further sequence analysis, we found that miR-141, miR-23a, and miR-27a may be involved in the downregulation of NF2 expression in breast cancer cells. Recent findings have shown that miR-20 regulates E2F1 negatively.^8,22^ Recently, Yu et al^17^ discovered a novel regulatory mechanism of breast cancer involving miR-20, which we also found here in PAOM 57.3. The following target genes have yet been verified by their miRNAs in breast cancer; however, the supporting evidences suggest that the relations are strongly associated with the breast cancer. CXCL12 expression is downregulated in primary breast carcinomas.^23,24^ Inactivation of the product of MAPK14 via PPM1D overexpression was also previously discovered in breast tumor cell lines.^25^ Supporting evidence for the role of NOTCH1 in breast cancer cells involves the fact that the rate of NOTCH1 expression in human breast cancer was found to be significantly higher than those of normal breast tissues at the margin of tumor sections.^26^ Zang et al^27^ showed that Notch signaling is overexpressed and highly activated in breast cancer. HMGA2 has been reported to be expressed in invasive and non-invasive breast cell lines.^28^ Yang et al^29^ experimentally verified that a core circadian *CLOCK* gene evidences tumor suppression properties and is downregulated in human breast cancer cells. Overexpression of THBS1 (TSP1) was detected in breast carcinoma and melanoma cells by interferon (IFN)-gamma-differentiated U937 cells in vitro via the release of reactive oxygen species.^30^ Finally, the expression of SERP1 was suppressed in papillary thyroid cancer.^31^ However, SERP1 and POLR2 have not yet been identified in breast cancer cells.

### Prediction of colon cancer

Table 2 shows the prediction of 15 relationships in colon cancer using the proposed method with filtering sequence analysis. HMGA2 (high mobility group [HMGI]) was observed to be abundantly expressed in human colorectal carcinomas.^32–34^ MAPK14 that is regulated by miR-124^11,35^ maintains a high level of ERbeta for E2 anti-proliferative effects in colon cancer cells^36^ and in giloblastoma.^37^ In addition, MAPK14 is involved in apoptosis in colorectal cancer induced by growth factors.^38^ The activation of the Wnt signaling pathway appears to suppress the expression of the *THBS1* gene in colon cancer cells.^39^ Jung et al^40^ observed that SIP1 (ZEB2), an E-cadherin transcriptional repressor, is induced by overexpressing TMPRSS2 in colon cancer cells, and affects the loss of E-cadherin-mediated cell-cell adhesion, resulting in an increase in cellular motility. Krugluger et al^41^ found that CLOCK is more abundantly expressed in colon cancer tissues than in normal tissue.

In addition, Kiriakidou et al^42^ experimentally reported that *CLOCK* is a target gene of miR-141. Recently, Zhang et al^43^ demonstrated that the Notch1 signal transduction pathway mediates the effect of COX-2 selective inhibitors on colorectal cancer cells, and also discovered the mechanism of the Notch1 pathway which regulates the proliferation and apoptosis of colorectal cancer cells. E2F3, a member of E2F family, is downregulated in the HCT 116 and RKO colon cancer cell lines.^44^ You et al^45^ determined that with the differential expression of dishevelled segment polarity protein 2 (DVL2), the Wnt signaling pathway may contribute to colon carcinogenesis. You et al^46^ reported that DVL2 was expressed in sporadic colon cancer tissues. Wendt et al^47^ recently reported that the expression of CXCL12 in human colorectal carcinoma cells reduced orthotopic tumor formation and inhibited metastasis in severe combined immunodeficient mice. HRAS and MTPN are yet to be confirmed.

## Discussion

Most previous computational studies have been conducted to predict miRNA-mRNA relations on the basis of DNA sequence data. The resultant large number of the sequence predictions makes biological validation quite difficult. On the contrary, a variety of previous studies have demonstrated that an miRNA deregulates its target mRNA in a cancer type-specific manner. For example, miR-34a deregulates E2F in cancer cell lines^44^ whereas miR-17 deregulates E2F in breast cancer cells.^20^ In this article, we suggested a PAOM consisting of a mathematical model and computational method using microarray data sets and filtering sequence analysis, such that the cancer-specific relationships between miRNAs and mRNAs can be predicted. The proposed PAOM was assessed and compared with normal and cancer microarray profiles in both breast and colon cancers. Among 676 relationships, we predicted 61 and 28 miRNA-mRNA relationships that might exert some effects on breast and colon cancer development, respectively. According to the results of sequence analysis filtering, we uncovered 18 breast putative relations with 12 mRNAs and 14 miRNAs and 15 colon putative relations with 12 mRNAs and 10 miRNAs. We confirmed that 8 genes—*MAPK14, CLOCK, NF2, HMGA2, CXCL12, E2F1, NOTCH1*, and *CD24*—are associated with breast cancer. Most importantly, we demonstrated that miR-20, a member of miR-17 cluster, is the target for E2F1 with PAOM score 57.3. Yu et al^17^ and Verghese et al^20^ independently verified that miR-20 regulates the development of breast cancer. In addition, Hossain et al^48^ confirmed that miR-17 down regulates E2F1 expression in breast cancer cells. Therefore, miR-20 is a strong candidate for targeting E2F1 mRNA in breast cancer. In particular, we predicted that CD24 is the target of let-7a and miR-98 with PAOM scores 48.2 and 12.0, respectively, in breast cancer,^19^ which was previously verified by Kaipparettu et al.^19^ Further studies will be necessary to verify those findings. In colon cancer cells, we predicted 15 relationships with 12 genes and verified 9 genes—*MAPK14, CLOCK, HMGA2, THBS1, CXCL12, SIP1, NOTCH1, E2F3*, and *DVL2*—influence on colon cancer. Overall, the proposed method described in this study was successful in the detection of some potential relationships, and may provide information for experimental studies targeting toward the identification of miRNA-mRNA relationships for specific cancers. However, there is no doubt that many unidentified relations continue to exist. Therefore, a novel approach using both computational methods and experimental validation is yet to be proposed for better outcomes in the prediction of miRNA-mRNA relationships in cancers.
